# Probable medications overuse headaches: validation of a brief easy-to-use screening tool in a headache centre

**DOI:** 10.1186/1129-2377-14-81

**Published:** 2013-10-02

**Authors:** Virginie Dousset, Martial Maud, Mélanie Legoff, Françoise Radat, Bruno Brochet, Jean-François Dartigues, Tobias Kurth

**Affiliations:** 1 Centre Douleur Chronique, Hôpital Pellegrin, Place Amélie Raba Léon, 33 076 Bordeaux, Cedex, France; 2 Inserm Unit 593, Neuroepidemioloy, Paris, France; 3 University of Bordeaux, Bordeaux, France; 4 Inserm Unit 708, Neuroepidemiology, Bordeaux, France

**Keywords:** Medications overuse headaches, Screening tool, Validation, Questionnaire, Sensitivity, Specificity

## Abstract

**Background:**

To validate a rapid questionnaire as a screening tool, because application of the diagnostic revised criteria of the ICHD-II for medication overuse headache (MOH) requires experience for the physician and is time-consuming.

**Methods:**

ICHD-II criteria for probable MOH (pMOH) were transformed in questions formulated in such a way that they could be self-administered, easily understood, and quickly filled out. We compared this questionnaire to the gold standard: the diagnosis made by headache specialists, based on the the ICHD-II criteria. Patients who were consulting for pMOH or migraine for the first time were consecutively included. As validity indicators, we calculated sensitivity, specificity, positive and negative predictive values of the items.

**Results:**

Seventy-nine patients were screened, 77 included, 2 female patients excluded. Forty-two patients have been considered as suffering from pMOH, 35 patients suffered from migraine without medication overuse. The association of the question “do you take a treatment for attacks more than 10 days per month” and the question “is this intake on a regular basis?” had a sensitivity of 95.2% and a specificity of 80%.

**Conclusion:**

This screening tool can detect pMOH with a sensitivity that could be of interest to screen patients in clinical practice and to pre-include patients for research as epidemiological studies.

## Background

The prevalence of chronic daily headaches with medication overuse is around 1.5% of the general population, with prevalence figures being very similar across different countries. They account for 10% to 78% of patients seen in headache centres [1]. Medication overuse headache (MOH) decreases the quality of life and accounts for reduced efficiency at work. Population-based and clinical data reflects the importance of this public health problem [2].

The 2nd edition of the International classification of headache disorders (ICHD-II) defined MOH as a migraine-type headache:1 present on ≥15 days per month with at least 1 of the following characteristics: bilateral, pressing/tightening quality, mild or moderate intensity; [3] associated with use of acute treatment for headache on a regular basis for more than 3 months [1] that developed or markedly worsened during medication overuse [4] that resolved or reverted to its previous pattern within 2 months after discontinuation of the offending medication [5]. Subsequent revision proposed to eliminate the headache characteristics [3] and to delete the last criterion in order to allow the establishment of MOH diagnosis when the patient suffers from the disorder and not only after the medication withdrawal [4]. This change suggested by Olesen has not been officially implemented and correct formulation for MOH before withdrawal should be probable MOH (pMOH). The IHS classification is an explicit clinical criterion so that the diagnosis of pMOH does not require any specific examination that is needed only to exclude a symptomatic origin. Application of the operational diagnostic revised criteria of the ICHD-II allows making the diagnosis on the basis of consumption of medication (number of days monthly) and a worsening of the headache during the time of medication intake. However, this clinical diagnosis with a face-to-face interview is the gold standard and requires clinical experience for the physician, and enough time to question patients precisely, to specify ICHD-II criteria, and to specify the diagnosis of primary headache, more frequently migraine, which has been the basis for the development of the MOH. This is time-consuming.

In this clinical context, a less time consuming tool would be of interest. We have developed a 4 item questionnaire evaluating the frequency of headaches and the amount of medication consumption used to treat the attacks and compared sensitivity and specificity of this in migraine patients and MOH patients. Indeed, such a tool would be useful in clinical practice and for research, to pre-include patients for epidemiological studies.

## Methods

### Design

We evaluated the accuracy of the rapid evaluation of headaches and medication used to treat the attacks consumption in a sample of consecutive headache patients seen at the Bordeaux Headache Centre for their first visit. Our goal was to establish the validity of a brief screening instrument that uses among other things self-reporting of use of acute treatment for headache by the patient and has both the sensitivity and specificity that would make it useful in the outpatient headache centres, and in general practitioner practices. The validity study required the comparison of answers of medication overuse headache sufferers to the answers of migraine sufferers, with the gold standard diagnosis done by two neurologists specialised in headache. The study was conducted in France in the Bordeaux headache centre. Because the setting of intended use was at first pain or headache centres, our validation study occurred in an outpatient headache centre (a specialized headache centre of a teaching hospital).

### Patients

All men and women aged 18 years or more who were visiting for migraine or probable MOH identified by their general practitioner were consecutively pre included. Pre inclusion required the ability to read and write French. They filled out the questionnaire in the waiting room. Then definitive inclusion was done after the diagnosis of migraine without aura or migraine with aura, or migraine without or with aura plus pMOH by one of the headache specialist during the face-to-face interview according to the 2004 ICHD-II [3] criteria.

Other primary headaches or secondary headaches were exclusion criteria. Patients were enrolled after providing their informed consent. We did not collect any personal information so that an ethics vote was not necessary.

#### ***Questionnaire elaboration***

The criteria defined pMOH as (A) headache present on ≥15 days per month, (B) associated with use of acute treatment for headache on a regular basis for more than 3 months ; (C) that developed or markedly worsened during medication overuse; (D) medication overuse is defined as 10 days or more of intake in triptans, ergot alkaloids, mixed analgesics or opioïds, and 15 days or more of analgesics/NSAIDs or the combined use of more than one substance. These criteria were transformed into questions by experts of MOH. They were formulated in such a way that they could be self-administered, quickly filled out and easily understood. The questions were: 1. Do you have headaches for more than 15 days/month? 2. Do you take treatment for attacks more than 10 days per month? 3. Is it for more than 3 months? 4. Is drug intake regular? (Table 1). The Item C of ICHD-II criteria (the headache worsened with the overuse of the acute medications) has been marked out because it’s a difficult question for the patients who answered about the intake of the medication to relieve the headache. In order to verify that all questions could be easily understood, we submitted the questionnaire to a sample of patients before beginning the study. Questions should have sufficient sensitivity to detect most patients with MOH and sufficient specificity to minimize the number of patients who screen positive for MOH but do not have the condition. This tool must not be time-consuming or burdensome for routine investigation in pain clinics. Before the face-to-face with the neurologist, the nurse of the headache centre gave the questionnaire to the patients. The first step of the study was that each of the patients filled out the questionnaire unaided, and unknown to the neurologist. Then the face to face consultation was realized, the neurologist didn’t know the answers of the patient to the questionnaire during the face to face interview. The same procedures were used in both groups.

**Table 1:** ICHD-II criteria for PMOH and self questionnaire

**ICHD-II criteria for probable MOH**	**Self-questionnaire**
**A.** Headache present on ≥ 15d/month	**1.** Do you have headache on ≥ 15 days per month?
**B.** Regular overuse for > 3 months	**2. Do you take a treatment for attacks on ≥ 10 days per month?**
1. Ergotamine, triptans, opioids or combination analgesic medications on ≥ 10 days /month on a regular basis for 3 months	**3.** For more than 3 months?
2. Simple analgesics or any combination of ergotamine, triptans, analgesics opioids on ≥15 D per month on a regular basis for > 3 months	**4. Is this intake on a regular basis?**
**C.** Headache has developed or markedly worsened during medication overuse	

The gold standard used to test the validity of the questionnaire was the face to face diagnosis of probable MOH made by headache specialists, based on the second edition of the ICHD-II. The gold standard procedure included a medical history, a comprehensive neurological history and examination. Possible organic causes of headache were excluded through a general and a neurological examination and if needed complementary exams. The headache specialist completed a symptom checklist based on ICHD-II criteria and assigned a clinical diagnosis of migraine or migraine plus MOH.

#### ***Validity assessment and data analysis***

Filling out the questionnaire and the face to face interview were done the same day. As validity indicators, we calculated sensitivity, specificity and the positive and negative predictive values for each pair, trio of item and for the 4 items. The sensitivity corresponded to the percentage of all affirmative answers in the group of patients with MOH (ability of detection of MOH cases). The specificity corresponded to the percentage of negative answers in the group of subjects with migraine (ability of detection of non-suffering MOH patients). The positive predictive value corresponded to the percentage of patients with MOH who screened positive. The negative predictive value corresponded to the percentage of patients without MOH who screened negative. Data analysis was done using SAS 8.2 for windows.

## Results

Patients were enrolled between September 2009 and December 2009. Eighty-nine patients have been seen for headache in the headache centre during this period.

Seventy-seven patients were pre-included, with twenty-one men (27.3%) and fifty-six women (72.7%). Ages varied from 16 to 71 years, with mean age of 42.5.

After being interviewed by the neurologists, 42 patients (55%) have been considered as suffering from probable MOH, 35 patient having migraine without medication overuse. There were more women with probable MOH than men (60.7% vs. 39.3% p = 0.08). The mean age in pMOH group was higher than in migraine group (46.7 (12.2) years vs. 37.4 (13.6) years, p = 0.002). Patients were heterogeneous concerning the duration of the disease and concerning the kind of medication they overused (Table 2).

**Table 2:** Age, sex and onset for migraine patients and pMOH* patients

	**Migraine**		**MOH**		**P value†**
	**n = 35**	**%**	**n = 42**	**%**	
Age (years)	37.4	13.6	46.7	12.2	**0.003**
Male	13.0	37.1	8.0	19.0	**0.060**
Female	22.0	62.9	34.0	81.0	
Onset (years)					
< 10 years					
>10 years	10.0	28.6	19.0	45.2	**NA**
	25.0	71.4	23.0	54.8	**NA**
Drug intake					
Acetaminophen or NSAID	2	25	6	17.6	
Combination	2	25	12	20.6	
Triptans	0		9	35.3	
Ergots opioids	1	12.5	0	26.5	
	2	25	12	35.3	

*probable Medication overuse headache.

†Comparison of means in student’s test.

Next we determined sensitivity, specificity, positive predictive value, negative predictive value for each item, for each pair of item and for the items 1 + 2 + 4.

The third question about the onset and duration of the drug intake was clearly not discriminating enough: 90.5% of patients have answered yes to the third question, that’s why item 3 has been marked out. Results are showed in Table 3.

**Table 3:** Sensitivity, specificity, positive predictive values, negative predictive values for each item and for more than one item

**Question**	**Sensitivity**		**Specificity**		PPV*		NPV**	
	**Absolute values**	**%**	**Absolute values**	**%**	**Absolute Values**	**%**	**Absolute values**	**%**
1	34/42	81.0	29/34	85.3	34/49	87.2	29/37	78.4
**2**	**40/42**	**95.2**	**26/34**	**76.5**	**40/48**	**83.3**	**26/28**	**92.9**
3	40/41	97.6	6/33	18.2	40/67	59.7	6/7	85.7
4	41/42	97.6	12/35	34.3	41/64	64.1	12/13	92.3
Yes for each question								
1 + 2	34/42	81.0	33/34	97.1	34/35	97.1	33/41	80.5
1 + 4	35/43	81.4	34/35	97.1	34/35	97.1	34/42	81.0
**2 + 4**	**40/42**	**95.2**	**28/35**	**80.0**	**40/47**	**85.1**	**28/30**	**93.3**
1 + 2 + 4	34/42	81.0	35/35	100.00	34/34	100.00	35/43	81.4

*positive predictive value.

**negative predictive value.

Concerning patients with pMOH, 19 patients had a duration of medication overuse > 10 years, 13 patients up to 10 years. Duration of pMOH didn’t have any influence on answers to questions. Twenty-four patients had pMOH type 1 as mentioned Saper and Lake [6], 18 type 2. This had no impact on answers. Eight patients overused acetaminophen or NSAID, 10 overused an association of medications, 14 overused combinations, 9 overused triptans and one ergotamine. The size of the sample did not allow to study the impact of the type of medication on the response profile. Duration of pMOH and classification of pMOH type 1 or 2 had no impact on answers to questionnaire (p = 0.07 and p = 0.01 respectively).

## Discussion

The main results of our study validation was the following: the association of question 2 (do you take a treatment for attacks more than 10 days per month?) and question 4 (is this intake on a regular basis?) had a sensitivity of 95.2% and a specificity of 80%. The advantages of such questions are their simplicity, rapidity, and low cost. Our sample of migraineurs, and of pMOH sufferers were heterogeneous in terms of age, sex, and duration. Moreover, we kept equivocal cases.

Several limitations of the results have to be discussed: At first, possible selection bias could exist: in a tertiary centre, patients could have a better knowledge of their disease, and could answer easier yes to the different questions, with better understanding, and therefore would be more able to quantify their medication intake. That could increase positive answers and then explain an increase of their sensibility in a tertiary centre. Moreover, patients seeking care in tertiary centres could have more severe disease, more severe medication overuse leading again to an increase in the questionnaire sensibility. For these reasons, generalisability to a population-based setting need to be further tested. Secondly problems with the measures have to be considered: concerning the construction of the questionnaire, the criteria specifying the necessity of having developed a worsening of the headaches during overuse was not kept. Indeed, patients usually answered that if they take medication, it is because they have headaches and not the opposite [7]. Patients usually think that medication overuse is the result of an unrecognized or inadequate treatment of a primary headache disorder. Moreover it is a retrospective question, about a period sometimes many years before the diagnosis of pMOH. However, to evaluate the reliability of transforming the criteria of having developed a worsening of the headaches during overuse , we did a pretest in a sample of 10 patients, asking them if they have developed worsening of the headaches during overuse asking them if they understood the question. The results of this pretest are shown in Table 4. Five patients answered that they didn’t develop a worsening of headaches during overuse, 2 patients answered that they did not know. That’s why this criterion was not kept. In the same way, we evaluated the ability to understand the other questions by the patients. Are expressions such as treatment for attacks intake on a regular basis easily understandable for patients without any medical knowledge? We did a second pilot study on a sample of 24 patients testing the comprehensibility of both these expressions: namely the expression treatment for attack was understood by 22 patients. The expression “intake on a regular basis” was understood by all, except one patient. At the end, if we do the hypothesis on a decrease of sensitivity in the general population, what could be the outcome of false positives, i.e. patients with an intake below 10 days per month, on the border of medication overuse: could it have negative implications for the patients wrongly classified as having pMOH by the questionnaire? In fact, it could have positive repercussions for the patients, with a primary or secondary preventive effect on the risk of developing pMOH. Concerning false negative patients, they will continue their overuse, until they see a neurologist or another physician. In our study, sensitivity and specificity could be high because of the sole inclusion of migraine sufferers, without including other primitive headaches, such as cluster headache and tension-type headaches. But, for a pilot study, it is acceptable to have included only migraine patients and CH frequency in tertiary centre only. What improvements does this questionnaire have in comparison to other published questionnaire: Table 5 presents data about the methods used in main epidemiological studies concerning MOH [5,8-13]. For each study, we specified the questions or criteria used for the MOH diagnosis, and specified if these questions were validated or not. One can notice that the only author using a validated questionnaire was Hagen [14]. In the general population he determined the diagnostic performances of the association of 2 questions: headaches which are present for more than 14 days per month and the use of analgesics 4 times per week or more. With such an association, a lower sensitivity was found: 75%, specificity was 100%. Other questionnaires do exist [14-17], but they concern research on dependence [15,16]. Radat et al. [15] constructed and validated a questionnaire measuring dependence on analgesics and on migraine treatments in headache patients. It measures with 21 questions a global dimension of dependence in MOH sufferers, but does not allow for giving a cut-off on which patients should be considered to have MOH. Such a dependence questionnaire which focus on the dimension of dependence are more time-consuming, and maybe more difficult to administrate in current care, neither in tertiary nor in primary care settings. Maizels in 2003 [18] proposed a paradigm for the clinical application of a brief headache screening tool. Questions were open and medication overuse was correctly identified in 86% of patients with a specificity of 79%. In this context, the advantages of this screening tool are its shortness, it’s easy administration without much paper work and low cost. We must consider its diagnostic performances outside tertiary centre, i.e. neurologists outside hospitals, general practitioners, and maybe for patients themselves outside of any health place. In particular, predictive values have to be determined in the general population. To conclude, we validated a screening tool in the French version for the diagnosis of pMOH. In the future, an English version should be validated.

**Table 4:** Answers to the question concerning the criteria specifying the necessity of having developed a worsening of the headaches during overuse: results of the pilot study in 10 patients suffering from pMOH

**Patient**	**Understanding of the question (Yes/No)**	**Answer to the question**
n° 1	Yes	No, because had tried to decrease the intake as early as the worsening of headaches
n° 2	Yes	Doesn’t know
n° 3	Yes	No
n° 4	Yes	Yes
n° 5	Yes	Yes
n° 6	Yes	No
n° 7	Yes	Doesn’t know
n° 8	Yes	Yes
n° 9	Yes	No
n° 10	Yes	No, because already tries to decrease drug consumption

**Table 5:** Methods used to diagnose MOH in main epidemiological studies

**Author**	**Country**	**Population**	**Questions or criteria**	**MOH Diagnosis**	**Type of questionnaire**
Castillo 1999	Spain	Random sample 2252 patients	Two questions:	Face-to-face diagnosis by a neurologist interested in headache	Unvalidated Questionnaire
			1- Have you ever had headache?		
			2- Do you have headache tend days or more per month?		
Wang 2000	Taiwan	Population of a small islet > 65 years-old	Revision HIS criteria proposed by Silberstein	Person-to-person survey method and diagnosis by a neurologist	Unvalidated Questionnaire
Lu 2001	Taiwan	Random population based survey	Face-to face questionnaire	Then face-to-face diagnosis with a physician	Unvalidated questionnaire
			Headache being present for more than 3 days per week in the previous year		
Colas 2004	Spain	Quota sampling approach > 14	Have you ever had headache?	Then face-to-face diagnosis with a physician	Unvalidated questionnaire
			Do you have headache 10 or more days per month?		
			If yes, to other questions: In the last three months have you taken any analgesic for your head pain		
Hagen 2009	Norway	All inhabitants of a county of Norway invited to answer to a questionnaire	Headache more than 14 days per month and use of analgesics 4 times per week or more	Then face to face interview by a neurologist	Validated questionnaire: sensitivity 75%, specificity 100%
Straube 2010	Germany	Adults from 3 German regions	14 single items designed to assess the 2nd ed of IHS, medication overuse classified based on medication intake during the last month.	Face-to-face by trained interviewers	Kappa statistics was calculated: 94.5% indicating excellent inter-rater agreement
Jonsson 2011	Sweden	Randomized cluster sampling	Two screening questions: headache present on ≥ 15 days per month and using medication for ≥ 10 days per month during the past three months	Then subsequent interview	Unvalidated questionnaire

## Competing interests

The authors declare that they have no competing interest.

## Authors’ contributions

VD carried out the study, MM participated to interpretation of data, ML and JFD participated in the design of the study and performed the statistical analysis. FR and BB participated in the design of the study. TK helped to draft the manuscript. All authors read and approved the final manuscript.
